# Emotional eating and weight regulation: a qualitative study of compensatory behaviors and concerns

**DOI:** 10.1186/s40337-018-0210-6

**Published:** 2018-09-14

**Authors:** Mallory Frayn, Simone Livshits, Bärbel Knäuper

**Affiliations:** Department of Psychology, McGill University, 2001 McGill College, Montreal, Quebec H3A 1G1 Canada

**Keywords:** Emotional eating, Normal weight, Eating behaviors, Compensatory behaviors, Qualitative research

## Abstract

**Background:**

Emotional eating, or overeating in response to negative emotions, is a behavior endorsed by both normal weight and people with overweight/obesity. For some individuals, emotional eating contributes to weight gain and difficulties losing weight. However, there are also many who engage in emotional eating who maintain a normal weight. Little is known about the mechanisms by which these individuals are able to regulate their weight.

**Methods:**

The present study seeks to gain insight into the behaviors of individuals of normal weight who engage in emotional eating through a series of one-on-one, 1-h long, qualitative interviews. Interviews were semi-structured and guided by questions pertaining to participants’ compensatory behaviors used to regulate weight and concerns regarding their emotional eating. All interviews were transcribed and then objected to a thematic analysis of their content.

**Results:**

The results of this analysis showed that participants endorsed using physical activity, controlling their eating behaviors, and engaging in alternative stress reduction and coping strategies to mitigate the effects of their emotional eating. They reported concern over the effects of emotional eating on their weight, body image, and health and saw this behavior as an unhealthy coping mechanism that was difficult to control.

**Conclusions:**

These results suggest that programs promoting exercise, mindful eating, emotion regulation, and positive body image could have a positive effect on emotional eaters who struggle to maintain a healthy weight.

## Plain English summary

Emotional eating is the act of eating in response to negative emotions and is commonly endorsed by individuals who have overweight/obesity, as well as those who are of normal weight. However, little is known about what allows individuals of normal weight to maintain their weight in spite of their emotional eating. The present study examined emotional eating in those of normal weight through a series of one-on-one interviews. The interviews asked questions about ways in which participants regulated their weight, as well as concerns held by participants about their emotional eating. Participants endorsed using exercise and regulating their eating behaviors in order to regulate their weight. They noted concern over their health and potential weight gain long-term. These findings have implications assisting emotional eaters who have overweight/obesity in helping them to regulate their weight and improve their health through exercise, mindful eating, and other stress reduction strategies.

## Background

Emotional eating is defined as the “tendency to overeat in response to negative emotions such as anxiety or irritability” ([1], p. 106). This behavior is of interest because emotional eating has been consistently associated with weight concerns such as overweight and obesity [2–4]. Additionally, individuals with overweight have been found to exhibit less effective coping skills in response to negative emotions, leading them to emotionally eat more frequently [5]. Difficulties with weight loss have also been associated with emotional eating (e.g., [6, 7]). Difficulties with weight loss that have been associated with emotional eating are increased binge eating, reduced self-monitoring, and lower quality social support [8–13].

Despite these findings, many individuals maintain a normal weight even though they engage in emotional eating [14]. Minimal focus has been dedicated to this group, and what differentiates these individuals from emotional eaters who become overweight or obese, as will be elaborated below. The present study seeks to illuminate the factors that allow emotional eaters of normal weight to maintain their weight through conducting in-depth one-on-one interviews. For reasons elaborated below, we chose to pursue the following two areas to increase understanding of the relationship between emotional eating and weight in individuals of normal weight: (1) compensatory behaviors used to regulate their weight, and (2) concerns regarding their emotional eating.

### Compensatory behaviors for weight regulation

The present study seeks to elucidate how some emotional eaters manage to maintain a normal weight despite consuming excess calories during emotional eating episodes. In order to do so, we aimed to identify behaviors that offset the excess calories (i.e. compensatory behaviors) that individuals of normal weight who engage in emotional eating use. Such compensatory behaviors may explain the lack of weight gain in these emotional eaters (e.g., [15–18]).

Many compensatory behaviors exist but specifically exercise and compensatory eating behaviors have been associated with emotional eating. Previous studies have shown that exercise contributes to weight maintenance in emotional eaters [1, 2], however the quantitative design of such studies has not allowed for an in-depth examination of what motivates these individuals to exercise and whether exercise is used as a direct compensation for overeating. Compensatory eating behaviors may also be related to emotional eating and weight maintenance. For example, the ability to monitor internal hunger and satiety cues has also been implicated in regulating eating behaviors and reducing food consumption [14, 19, 20]. However, specific ways in which emotional eaters of normal weight may rely on these cues to regulate their eating and weight is not known.

#### Exercise as a compensatory behavior

Engaging in regular physical activity has been shown to protect against weight gain [21] as well as reduce depression and other forms of negative affect [22] that can lead to emotional eating. Subsequent longitudinal studies have shown the importance of physical activity for emotional eaters; physical activity has been found to moderate the relationship between emotional eating and weight gain over time [4]. Dohle et al. [2] similarly found that emotional eaters who exercised more frequently had lower BMIs over a 1-year period than those who exercised less frequently. Physical activity may help compensate for overeating and thus help to prevent weight gain typically observed in emotional eaters.

#### Compensatory eating behaviors and emotional eating

Emotional eating has been associated with reduced awareness of internal hunger and satiety cues, in part because stress alters one’s ability to be aware of these internal cues [19, 20]. also found that individuals who experienced binge-eating episodes engaged in emotional eating because they were unable to suppress their food consumption. However, individuals of normal weight who engage in emotional eating have been found to consume less food in response to negative emotions than individuals with overweight or obesity [14]. Thus, it may be that individuals of normal weight are more aware of their internal hunger and satiety cues even under stress.

Other compensatory eating behaviors are associated with eating disorders and disordered eating behavior. For example, fasting is a correlate of body dissatisfaction, internalization of thin ideals, and restrained eating [23]. Purging is also associated with eating disorders including bulimia and binge eating disorder [24]. The present study seeks to examine whether or not such behaviors are present in emotional eaters of normal weight.

### Concerns regarding emotional eating

Emotional eating has been related to concerns including increased external motivation for eating healthily and heightened monitoring of one’s food consumption, outside of emotional eating episodes [25]. Similarly, worries about weight prior to a physical activity intervention have been shown to predict emotional eating and continued concern about weight but not post-intervention BMI [26]. Thus, weight concerns may help to protect against actual weight gain. The present study aims to identify what concerns individuals of normal weight who engage in emotional eating may have with regards to their eating behaviors and explore how such concerns may motivate weight regulation.

#### Concerns regarding body image

Negative body image has been associated with emotional eating [27]. Additionally, being discontent with one’s body is related to wanting to lose weight [28]. Conversely, individuals who are less concerned about their body image and eating habits may be less likely to engage in emotional eating [29]. Individuals with greater flexibility in their body image are also less likely to binge eat, which is associated with emotional eating [30]. However, previous research on the relationship between body image, weight, and emotional eating has predominantly studied individuals with overweight or obesity or individuals with diagnosed eating disorders. The present study will explore the extent to which individuals of normal weight who engage in emotional eating experience body image concerns and examine the relationship between these concerns, emotional eating, and weight.

### Aim

Through qualitative interviews examining the compensatory behaviors and concerns associated with emotional eating in individuals of normal weight, the present study aims to elucidate the mechanisms through which individuals of normal weight who engage in emotional eating maintain their weight.

## Methods

### Participants

Participants for the study were undergraduate students recruited from a larger survey study (*N* = 200) exploring the relationship between emotional eating and several psychological constructs. Both studies were approved by the university’s research ethics board. Of those who completed the survey, a total of 58 participants were deemed eligible to participate in the present study based on the following three inclusion criteria: (1) scoring 3.25 or higher on the emotional eating subscale of the Dutch Eating Behavior Questionnaire (DEBQ; [31]), which represents the 80th percentile based on a normative Dutch sample [32], (2) endorsing a BMI within the normal weight range based on self-reported height and weight, and (3) reporting that they had maintained their weight within 5 pounds over the past 1 to 2 years. Weight maintenance was a criterion to ensure that participants were not formerly of higher weight, and thus did not differ in compensatory behaviors and concerns based on previous weight status.

### Sampling

Recruitment for the present study was conducted sequentially, as the larger survey study from which participants were selected was ongoing. For every 50 participants who completed the larger survey study, eligible participants were identified based on the criteria outlined above. Two participants were then randomly selected using a random number generator (randomizer.org) and sent an email invite to participate in the interview. If a participant failed to respond, a new one was randomly selected and contacted from the same cohort of eligible individuals. Throughout this process and until data saturation was achieved, a total of five individuals refused to participate (3 did not respond to the initial email, 1 stated that they were too busy to participate, and 1 failed to attend their interview appointment with no reason provided). This initial email was the only direct contact participants had with the researcher prior to the study.

### Data saturation

There is a lack of consensus between researchers regarding the number of interviews required in qualitative research to reach data saturation [33]. Rather it has been suggested to treat data saturation as a moving target that is achieved once no new codings and themes are identified from additional interviews [34]. To facilitate this approach in the present study, participants were recruited two at a time and preliminary codings and themes were generated after the first couple of interviews and then refined as subsequent interviews were conducted. Recruitment was terminated when new themes ceased to emerge in interviews. This point of data saturation was achieved after eight interviews in the present study as per consensus amongst the three researchers (all authors). This number of interviews was in line with findings by Guest, Bunce, and Johnson [34] who conducted an experimental study and found that data saturation may occur within as few as six interviews. As emphasized by Burmeister and Aitken [35], the focus of the present study was on data richness and depth, rather than solely data quantity.

### Measures

The DEBQ is a self-report questionnaire with 33 items on three different subscales: restrained eating, emotional eating, and external eating. For the purpose of this study, only the 13-item emotional eating subscale was used. Participants were asked to rate the frequency with which they experienced the desire to eat in response to a variety of emotions on a 5-point Likert-type rating scale from never (1) to very often (5). The DEBQ has been found to have high factorial validity as well as high internal consistency [31].

### Procedure

Individual in-depth interviews were conducted with each participant to learn about various aspects of their emotional eating. Each interview lasted between 45 to 60 min and was conducted in a closed office on the university campus. Participants were compensated $20 for their time at the end of the interview. The interviews were semi-structured, following a set of questions examining three aspects of emotional eating: (1) history of emotional eating, (2) compensatory behaviors used to maintain weight, and (3) concerns regarding emotional eating. For the purpose of this study and its focus on emotional eating and weight regulation, only data from the latter two sections of the interview were included. The interviews were conducted by the first author, a female senior PhD student in clinical psychology who had received training in interviewing and assessment as part of her clinical training. A female undergraduate research assistant (second author) observed some of the interviews for learning and training purposes.

Prior to commencing each interview, the researcher introduced herself and provided participants with a brief overview of the purpose of the study, namely that the researchers were interested in examining the relationship between emotional eating and weight. Participants were informed that they would be asked several questions pertaining to this topic and that they were free to answer as they wished, both regarding content and level of disclosure. They were also informed that the interview would be audio recorded for transcription purposes. All participants consented to this prior to study commencement.

The section on compensatory behaviors explored strategies that participants utilize to regulate their weight. They were asked how they thought they maintained a normal weight despite their eating behaviors, as well as what behaviors they actively engaged in to maintain their weight. Questions were also asked to better understand what their episodes of emotional eating looked like, delving specifically into the factors that caused them to stop eating.

The final section asked questions about participants’ concerns regarding emotional eating. This part of the interview explored the feelings individuals had when engaging in emotional eating, as well as the possible negative effects they thought emotional eating might have on their life, both in terms of weight and other health problems. Questions were also asked to ascertain individuals’ interest in reducing or eliminating their emotional eating, versus how they felt about continuing to engage in this behavior.

## Results

### Data analysis

Braun and Clarke’s [36] procedure for thematic analysis in psychological research was used to analyze the data. A data-driven approach was used for the analysis with themes derived from the data itself instead of being identified in advance. All interviews were first transcribed and then coded into basic elements. Codings were then organized into preliminary themes, based on the sections of the interviews pertaining to compensatory behaviors and and concerns with emotional eating. Preliminary themes were reviewed and edited, prior to defining and naming a finalized list of themes that encompassed the entirety of the data set. Codings and themes were reviewed by two researchers (first and second author) throughout the analytic process to ensure consensus.

### Demographics

A total of 8 participants were interviewed, 7 of which were female. 88% of the sample identified as Caucasian. The mean age of participants was 19.00 years and the mean BMI was 22.09. Participants scored an average of 3.78 on the DEBQ emotional eating subscale.

### Themes pertaining to compensatory behaviors

Four themes were identified regarding compensatory behaviors used by individuals to compensate for their emotional eating: (1) physical activity as a compensatory behavior, (2) the use of alternative stress reduction and coping strategies, (3) compensatory eating behaviors, and (4) the impact of metabolism.

#### T1: Physical activity as a compensatory behavior

The vast majority of participants endorsed the use of physical activity to compensate for their emotional eating and regulate their weight. The type and duration of physical activity varied between participants, with some participants engaging in unstructured, moderate exercise (e.g., long walks) and others reporting structured, high intensity exercise (e.g., cardio exercises such as running or interval training). Some participants noted that they engaged in physical activity regardless of the severity and frequency of emotional eating episodes while others described engaging in more physical activity after episodes of emotional eating. Multiple participants reported using exercise for stress relief to avoid emotional eating.

*I know that like as long as I get a workout in before noon every day, the rest of my day is going to be great. It’s going to be fine, and whatever stress I have, I’m not going to go to an extreme. (8).*

Some participants also connected their use of physical activity to helping alleviate mental health concerns, both in the presence and absence of their emotional eating.

*Even when I’m not overeating, exercise just makes me feel like a lot better, physically, but also emotionally and mentally. (4).*

#### T2: The use of alternative stress reduction and coping strategies

Participants cited the use of specific stress reduction techniques and other coping strategies as replacements for the mood enhancing effects of emotional eating. Such techniques included tools derived from Cognitive Behavioral Therapy (CBT), like thought records for cognitive restructuring, that participants described learning during therapy for mental health concerns such as anxiety. Participants who had experienced mental health concerns noted that managing these concerns helped them to reduce emotional eating.

*Part of the CBT techniques that my counsellor taught me was to pinpoint exactly what triggered my bad mood, and work from there to see whether or not it’s rational for me to be upset over it, or if I’m blowing things out of proportion. (1).*

Social support was also mentioned as a coping strategy. Some participants said that engaging socially with others compensated for negative emotions, such as loneliness, that led to emotional eating. Multiple participants discussed talking to friends about things that were bothering them while others reported using their parents as a support system.

*It feels like I don’t have to just turn to food to feel better, I can turn to friends instead. (7).*

#### T3: Compensatory eating behaviors

Participants described eating behaviors they engaged in after emotionally eating to compensate for their overconsumption. A common theme was the reduction of food intake after emotional eating. Some participants fasted in the days after emotional eating episodes while others simply ate less food in the subsequent days.

*If I do have a big weekend of eating, like a big emotional eating session, I will be more careful in what I’m eating for the following days. (6).*

Many participants also endorsed the desire to engage in healthy eating habits, regardless of their emotional eating, viewing it as a lifestyle choice. However, many also cited healthy eating as motivation to avoid emotional eating. For example, participants described that by starting their day in a healthy way, they were more likely to continue eating healthy (and thus avoid emotional eating) throughout the remainder of the day.

Some participants took their perception of “healthy” eating to an extreme, engaging in cleanses after prolonged emotional eating. Most, however, simply monitored what they consumed and elected to make healthy, balanced dietary choices. Several participants also endorsed vegetarian or vegan lifestyles, which required them to consume healthier foods.

*A big thing that has helped me in changing my diet has been becoming a vegetarian, and now becoming a vegan. I kind of create even more restrictions to my day. (5).*

Several participants mentioned that they avoided overeating during emotional eating episodes. In other words, despite consuming unhealthy foods when emotional, many described still trying to stop once they noted that they were full.

*I don’t tend to overeat that much because I don’t want to gain weight. I don’t want to be overweight, so I’ll overeat to like 5% past my capacity. I won’t get to a point where I want to vomit, it’ll just be a point where I’m full. (3).*

Some participants noted mindful eating habits; they described intuitively paying attention to their hunger and satiety cues to guide their eating. This body awareness was attributed to a few factors. One participant credited chronic pain with helping her to be aware of what her body needed, while others endorsed feeling in tune with their bodies as helping them maintain a normal weight.

*I’ve become a little bit better with recognizing what it is my body needs {as a result of chronic pain}, and this awareness helps with my eating. (4).*

Avoiding unhealthy trigger foods was another strategy frequently used by participants. Participants endorsed not buying certain foods that they knew they would be likely to consume in response to emotions. Some participants avoided grocery shopping while hungry as to not make unhealthy choices, or even hid food from themselves to avoid consuming it while emotional.

*Peanut butter, Nutella, those are my two big ones. So those just don’t come into my apartment, and if they do they’re in little individual packages, because it’s really hard to eat those without noticing. (6).*

Notably, most people did not endorse purposefully purging to compensate for overeating. One participant noted that while they did not actively attempt to purge, they would often eat so much during emotional eating episodes that they would inadvertently vomit.

*I’m often physically sick like 75% of the time {when I engage in emotional eating}. (6).*

Finally, participants put forward the idea that avoiding emotional eating behaviors led to feelings of competence and autonomy. In other words, avoiding emotional eating appeared to increase participants’ self-efficacy that they could continue to disengage from this behavior and engage in healthier behaviors instead. Some participants thus made active attempts to improve their emotional eating habits and become healthier, as well as to attain a more balanced lifestyle.

*I’ll start, and like, today’s going to be different, today I’ll have a healthy breakfast, and then once you do and you feel really good about it and you’re like “hey, this is nice to maintain”, and then yeah, I feel like it’s also just like a meal prep kind of thing of like “oh, I’ll make this and then I’ll have it for lunch today, and lunch tomorrow, and then I’ll take this snack to my class”. (5).*

#### T4: The impact of metabolism

Several participants believed that they were able to maintain their weight because of a fast metabolism. These participants, more often than not, reported that they did not eat particularly healthily and also did not exercise. However, they did acknowledge that they would not always be able to rely on their metabolism to maintain their weight.

*I honestly could not tell you {how I maintain my weight}. I find it a miracle that I’m not morbidly obese. I think it’s probably some sort of genetic thing because my weight doesn’t fluctuate. (4).*

### Themes regarding concerns about emotional eating

There were six overarching themes regarding participants’ concerns about their emotional eating: (1) concerns about weight, (2) concerns about health, (3) emotional eating as an ineffective coping mechanism, (4) emotional eating as difficult to abate, (5) avoiding immediate negative physical and psychological effects of emotional eating, and (6) negative social evaluation.

#### T1: Concerns about weight

The majority of participants endorsed concerns about eventual weight gain. While some participants viewed emotional eating as a barrier to attaining their ideal body weight, others believed that over time, emotional eating would cause them to become overweight. Some participants put forward the idea that their worry about weight gain would protect them from actually gaining weight. Similarly, some participants noted that they were diligent about compensatory behaviors such as exercise because they were concerned about weight gain.

*I still do have a lot of anxiety over weight gain, so when I do have a large emotional eating session, I think about that a lot and stress over it, which is one of the reasons why exercise is such a compulsion afterwards. (8).*

Although many participants were more concerned about long term weight gain, some participants endorsed that their emotional eating could trigger them to worry about immediate weight gain.

*Total regret. Yeah, as soon as I start eating it, I’ll be like “Ah this was a mistake”. I know it’s not happening, but I feel myself physically gaining weight. (3).*

Additionally, a few participants described a relationship between avoiding emotional eating and body image concerns. The negative body image that they believed would come with weight gain was cited as motivation to avoid emotional eating.

*I’ll always have that fear of putting that weight back on, so that also keeps me from doing it a lot. I was just so unhappy at the weight that I was, I wasn’t comfortable in my body, I didn’t feel pretty, I hated my body. I’m terrified of ever feeling like that again. (6).*

#### T2: Concerns about health

Participants reported concerns about their health, regardless of weight. Multiple individuals noted that they were actively trying to reduce their emotional eating because of anticipated health concerns. Some described worry about experiencing similar health concerns to their parents, such as developing chronic diseases like diabetes. Participants mostly predicted long-term concerns about their health but were not noticeably concerned about the implications of emotional eating on their health in the short-term. Multiple participants also noted that they were concerned about health problems associated with weight cycling that could occur as a result of emotional eating. Regardless of weight gain, however, individuals noted concern about the potential effects of their emotional eating on their overall health.

*Even though you’re not putting on weight, it still can affect your cholesterol, your, you know, everything else. There could be health consequences, so there’s that that you need to be mindful about also. (4).*

#### T3: Emotional eating as an ineffective coping mechanism

Some participants viewed emotional eating as an unhealthy way to cope with their problems. These participants believed that emotional eating carried mental repercussions such as negative body image and ineffective coping. A few participants put forth the idea that emotional eating covered up a deeper issue that needed to be dealt with. Some of the participants who endorsed emotional eating as an unhealthy way to cope with stress reported that they were actively working on using alternatives to coping mechanisms.

*I think it’s kind of a cover-up to a deeper issue that you’re not dealing with. You have an issue and instead of learning to deal with it, you’re covering it up. While that might work for the time, you can’t live your whole life avoiding your problems. At some point, something’s going to catch up to you. (5).*

Multiple participants cited concern that their emotional eating would lead to other, more problematic behaviors. They believed that engaging in emotional eating reduced their willpower and could make it easier to use other substances for comfort and emotion regulation. In other words, they cited concerns about “addiction transfer” from food to other addictive substances.

*I think it’d just become more and more easy to turn to any substance that would make you feel better. If it’s not food, it’s cigarettes, it’s drugs, it’s drinking. I think eventually, you just start looking for something to make you feel better, and then that stops working, so you look for something better than that, and something better than that. It can definitely be a spiral. (6).*

#### T4: Emotional eating as difficult to abate

Participants were varied in their motivation to cease emotional eating. Many participants believed that their emotional eating would be virtually impossible to get rid of. While some described that they were actively trying to reduce emotional eating, others were more ambivalent about changing their emotional eating. Multiple participants tended to normalize their emotional eating, justifying that because they were normal weight, they needed not be concerned about it. Some had previously tried to eliminate their emotional eating and because of failed past attempts they were now content with the reality that their emotional eating could not be eliminated.

*I mean, it’s always good to dream that it will go away, but knowing myself I’ll know that I’ll be able to reduce it, but it will never go away. It will always be this part of me and it’s just going to come back. (7).*

Many participants described concerns pertaining to emotional eating and control. Control was described on a continuum from feeling in control of their emotional eating at times, to worrying about “losing control” over emotional eating. For many of the participants who described concerns with control, emotional eating was considered an addiction. Also, some participants felt ashamed of their emotional eating and regarded it as an indicator of low self-control.

#### T5: Avoiding immediate negative physical and psychological effects of emotional eating

Most participants described that both the physical and psychological effects that occurred as a result of emotional eating were unpleasant. Some participants noted that they disliked the bloated and lethargic feelings that resulted from overeating. Participants also endorsed that avoiding aversive physical consequences related to emotional eating motivated them to avoid engaging in this behavior. Some participants said that they avoided emotional eating because they knew that their bodies felt better when they consumed healthier foods.

*Throughout the years with exercising more and everything, I feel like I have become a little better at recognizing what’s healthy and what’s not. As I’ve started eating healthier, my body just doesn’t react as well to junk food. (4).*

Participants also cited the desire to avoid aversive psychological consequences of emotional eating, such as feelings of guilt and shame. Many participants described that guilt helped them to self-regulate. For example, for some participants guilt arose from fear of gaining weight, thus motivating them to avoid emotional eating. Overall, participants described that negative psychological feelings such as guilt helped motivate them to not engage in emotional eating.

*I don’t do well with guilt generally, and I generally tend to build up a lot of guilt that’s unnecessary. So if I feel guilt after emotional eating, it really hits me hard, and I feel like it’s motivation that I don’t want to feel bad about this again. (5).*

Conversely, other participants endorsed that they did not experience negative feelings such as guilt after emotionally eating. They reported feeling that emotional eating was normal, had no noticeable effects on their body, and that the act of eating palatable food was overall pleasant.

*It’s anticipation {of eating}, it’s enjoying it at the time. I don’t know if I would necessarily feel super guilty after, maybe because it’s become kind of status quo and I accept a little bit that it’s out of my control. (2).*

#### T6: Negative social evaluation

Several participants saw their eating habits as abnormal compared to that of their peers and cited this as a motivation to change their behavior. Hearing other people’s negative comments about their eating, especially those of family members helped some participants reduce their emotional eating. Others described that seeing their roommates and friends eating healthier foods motivated them to do the same and thus not engage in emotional eating behaviors.

*I feel like there’s more of an expectation living with roommates. When I see them going through a healthy day, it’s like, “well I’m not even hungry, so why, when like they’re not constantly eating, why do I?” Then I don’t do it as much, because you see healthy behavior and you’re like, “well, that seems more logical, I’m going to do that.” (5).*

Others endorsed the concern that their behavior would be off-putting to others if they were aware of it.

*It was always the feeling of getting caught, and just being embarrassed that somebody saw me in that state, so, not that I think there’s a real consequence, but more I just fear that judgment again. (6).*

## Discussion

The present study aimed to provide insight into the ways in which individuals of normal weight who engage in emotional eating are able to maintain their weight, approaching this goal through qualitative interviews. These interviews focused broadly on two domains: (1) compensatory behaviors used by these individuals to maintain their weight, and (2) concerns held by individuals of normal weight regarding their emotional eating.

### Compensatory behaviors

Physical activity was a compensatory behavior frequently endorsed by participants. Because physical activity has been found to protect against weight gain, this relationship may explain why participants were able to maintain their weight while emotionally eating [2, 4, 21]. Additionally, participation in physical activity may play a role in reducing the severity of mental health concerns and disordered eating that were associated with emotional eating in the present study. Herring, O’Connor, and Dishman [37] conducted a systematic review finding that exercise improved symptoms of anxiety. Because emotional eating has been linked to mental health concerns such as anxiety and depression [38], these findings provide empirical support that physical exercise may have alleviated some of these concerns and thus lessened the severity of emotional eating for some participants.

Several participants mentioned their metabolism as a key factor in their weight regulation, attributing their ability to maintain a normal weight to their genetics and ability to metabolize foods quickly. Interestingly, these participants were less likely to endorse actively using compensatory mechanisms to try to maintain their weight. Future research could examine whether such individuals are more likely to gain weight in the future given that metabolic processes slow during aging [39].

Despite regularly engaging in emotional eating, some participants also endorsed the use of alternative stress reduction and coping strategies to try and reduce the frequency of their emotional eating. It is likely that the development of such strategies assisted their weight regulation as these participants had alternative options to cope with negative emotions that did not involve food. This finding points to the importance of teaching emotion regulation to emotional eaters to promote both physical and mental health.

Additionally, several participants cited compensatory eating behaviors; these individuals endorsed some ability to suppress their food intake in attempts to avoid overeating. Thus some individuals of normal weight who engage in emotional eating may be able to maintain their weight because they minimize consumption of large food portions during emotional eating episodes. This regulation of intake may result from attending to their internal hunger and satiety cues, as was cited by participants in the present study, and is consistent with the findings of Tan and Chow [20]. Thus listening to internal cues to moderate food intake may help facilitate weight maintenance in emotional eaters of normal weight.

### Concerns

Participants frequently endorsed concerns about their weight, specifically citing worry about weight gain. Consistent with previous research, these individuals endorsed high worry regarding weight gain, despite emotional eating not influencing their actual weight [26]. While body image concerns have been related to a desire to lose weight [28], participants in the present study cited motivation to maintain their current weight to avoid negative body image in the future. Participants also described concerns pertaining to their future health. This finding is consistent with studies that have found that emotional eating may lead to concerns such as heightened monitoring of eating habits and greater external motivation to pursue healthy eating and lifestyle habits [25].

Furthermore, participants tended to view their emotional eating as an ineffective coping mechanism and also cited concerns that their behaviors could lead to negative social evaluation. It is possible that some individuals in the present study experienced stigmatization due to their emotional eating. Experiencing stigma has been associated with having eating disorders such as binge eating disorder [40], which is related to emotional eating. Further research is needed to explore the presence of stigma towards emotional eaters and the possible effects this may have on these individuals.

Finally, participants endorsed the idea that emotional eating was difficult to abate, despite attempts to engage in alternative forms of emotion coping. They also noted that they disliked and thus attempted to avoid the short-term negative physical and psychological effects of emotional eating, such as guilt and shame, as much as possible. This is consistent with the findings of Bennett, Greene, and Schwartz-Barcott [41] who found that guilt was associated with emotional eating, especially in females. Despite maintaining their weight, perceived weight concerns held by individuals of normal weight who engage in emotional eating may lead them to use food to cope, similar to those with overweight and obesity.

## Conclusion

Findings from the present study highlight ways in which emotional eaters of normal weight maintain their weight. Comparing the present findings with the literature on emotional eating in individuals with overweight/obesity can provide insight on ways to target emotional eating in individuals of various weight statuses. For example, some emotional eaters in the present study were found to consume what they described as small amounts of food in response to negative emotions. Past studies have found that individuals with overweight and obesity consume greater amounts of food during negative mood-inducted emotional eating episodes than individuals of normal weight [14, 42]. In the present study, efforts to regulate food consumption was related back to both awareness of hunger and satiety cues, as well as attempts to use alternative coping strategies to address negative emotions. Thus emotional eaters of all sizes may benefit from learning strategies for regulating food intake, such as mindful eating techniques e.g., [43]. These techniques may help them to better attend to their internal hunger and satiety cues to guide them in when and how much to eat. Programs that involve emotion regulation strategies would also be useful, such as those that teach emotional eaters how to utilize healthier coping mechanisms like social support and self-care when they are experiencing negative emotions. Therapeutic approaches such as Acceptance and Commitment Therapy (ACT; [44]) and Dialectical Behavior Therapy (DBT; [45, 46]) may be applied to help promote distress tolerance in emotional eaters.

Also, these results suggest that promoting exercise may be useful for emotional eaters, both in terms of weight regulation and stress reduction. As discussed, past studies have found a protective effect of exercise on weight gain in emotional eaters [2, 4]. However, it is also necessary to consider the way in which exercise is viewed by the individual before recommending it to target emotional eating. In the present study, several participants used exercise to compensate for their emotional eating in an almost compulsive manner, exercising excessively to burn off perceived excess calories. This type of exercise has been implicated in disordered eating behaviors [47]. To avoid this, disordered eating should be screened for and exercise should be tailored to the individual, including both psychoeducational and nutritional information [48].

Finally, because negative body image has been associated with emotional eating, both by individuals of normal weight in the present study and by those with overweight and obesity in past research [27], programs that target body image improvement could also improve the overall health and well-being of emotional eaters.

### Limitations and future directions

This study has a few limitations that should be addressed. First, the majority of the participants interviewed were Caucasian women. Additionally, all of the participants were undergraduate students, i.e. young adults and highly educated. Given the homogeneity of the sample, it is important that future research target other populations of individuals of normal weight that engage in emotional eating. The findings of this study point to future directions for research on emotional eating, such as further examining differences between emotional eaters who are normal weight versus those with overweight and obesity in self-regulation, fear of weight gain, and body image concerns.
